# Fast and Uncooled Semiconducting Ca-Doped Y-Ba-Cu-O Thin Film-Based Thermal Sensors for Infrared

**DOI:** 10.3390/s23187934

**Published:** 2023-09-16

**Authors:** Annick Dégardin, David Alamarguy, Aurore Brézard Oudot, Samir Beldi, Christine Chaumont, Faouzi Boussaha, Antoine Cheneau, Alain Kreisler

**Affiliations:** 1 Université Paris-Saclay, CentraleSupélec, CNRS, Laboratoire de Génie Électrique et Électronique de Paris, 91190 Gif-sur-Yvette, France; 2 Sorbonne Université, CNRS, Laboratoire de Génie Électrique et Électronique de Paris, 75005 Paris, France; 3 ESME Research Lab, 38 rue Molière, 94200 Ivry-sur-Seine, France; 4 GEPI, Observatoire de Paris, Université PSL, CNRS, 75014 Paris, France

**Keywords:** Y-Ba-Cu-O semiconductor, calcium doping in Y-Ba-Cu-O, amorphous thin films, uncooled near-infrared sensors, pyroelectric detectors, noise measurements, detectivity, fast pyroelectric response

## Abstract

YBa_2_Cu_3_O_6+*x*_ (YBCO) cuprates are semiconductive when oxygen depleted (*x* < 0.5). They can be used for uncooled thermal detection in the near-infrared: (i) low temperature deposition on silicon substrates, leading to an amorphous phase (*a*-YBCO); (ii) pyroelectric properties exploited in thermal detectors offering both low noise and fast response above 1 MHz. However, *a*-YBCO films exhibit a small direct current (DC) electrical conductivity, with strong non-linearity of current–voltage plots. Calcium doping is well known for improving the transport properties of oxygen-rich YBCO films (*x* > 0.7). In this paper, we consider the performances of pyroelectric detectors made from calcium-doped (10 at. %) and undoped *a*-YBCO films. First, the surface microstructure, composition, and DC electrical properties of *a*-Y_0.9_Ca_0.1_Ba_2_Cu_3_O_6+*x*_ films were investigated; then devices were tested at 850 nm wavelength and results were analyzed with an analytical model. A lower DC conductivity was measured for the calcium-doped material, which exhibited a slightly rougher surface, with copper-rich precipitates. The calcium-doped device exhibited a higher specific detectivity ($D^{*}=7.5\times 10^{7}\;cm\cdot \sqrt{Hz}/W$ at 100 kHz) than the undoped device. Moreover, a shorter thermal time constant (<8 ns) was inferred as compared to the undoped device and commercially available pyroelectric sensors, thus paving the way to significant improvements for fast infrared imaging applications.

## 1. Introduction

Two main families of physical principles are commonly implemented for detection of infrared (IR) radiation: quantum detection and thermal detection. 

In a quantum detector or photodetector [1], a semiconductor, as active material exposed to IR radiation, absorbs photons of sufficient energy (equal to or larger than the material bandgap); this creates electron-hole pairs increasing the material conductivity (photoconductors) or generating a photocurrent proportional to the incident radiation power (e.g., reverse-biased photodiodes, phototransistors). It should be noted that nanomaterials (e.g., colloidal quantum dot phototransistors [2]) or two-dimensional (2D) materials (e.g., graphene photodetectors [3,4]) lead to the development of highly sensitive and very high-speed quantum detectors.

In thermal detectors, the incident radiation absorption induces a temperature variation of the sensing element leading to measured changes in its physical/electrical properties: e.g., a variation of the electrical resistance for bolometers [5]; a variation of the electrical polarization for pyroelectric detectors [6]; the generation of a voltage for thermopiles [7]; or the thermal expansion of a gas for Golay cells [8]. Since they respond to temperature variations, thermal detectors exhibit the inherent drawbacks of a slow response (typically in the few tens or hundreds of milliseconds to few tens of microseconds range) and a relatively low responsivity as compared to quantum detectors. However, they also exhibit the main advantage of a wide spectral response compared to quantum detectors. In addition, thin film-based technologies in the field of uncooled IR sensors offer lightweight, compact, low-power, and low-cost detector systems. 

At the core of the uncooled thermal detector development is the material choice for sensing the incoming radiation. Various materials have been used for developing thin-film pyroelectric detectors [9]: e.g., P(VDF-TrF) copolymers [10], lead zirconate titanate (PbZrTiO_3_ or PZT) [11], lithium tantalate (LiTaO_3_) [12], and lithium niobate (LiNbO_3_) [13], with good performances. For instance, the LiTaO_3_ sensor published in [12] exhibited a good detectivity value $D^{*}=$ 1.7 × 10^8^ cm·Hz^1/2^·W^−1^ at 80 Hz but a slow response (thermal time constant $\tau_{th}=$5.8 ms). Other materials have also been explored for their pyroelectric response, such as aluminium nitride (AlN) [14,15] or yttrium–barium–copper oxide (YBa_2_Cu_3_O_6+*x*_, hereafter called YBCO) [16,17]. 

YBa_2_Cu_3_O_6+*x*_ is well known as a high-critical-temperature superconductor for an oxygen content *x* higher than 0.7. This superconducting phase has been largely explored for developing cooled highly sensitive hot electron nanobolometers for terahertz waves [18,19]. But YBCO also exhibits a semiconducting phase when oxygen depleted (with *x* < 0.5), which has been explored for the development of pyroelectric detectors exhibiting high performances [20,21,22]. Indeed, semiconducting YBCO offers key advantages in the field of uncooled IR detection: (i) film deposition at low temperature (below 200 °C), leading to amorphous films (hereafter called *a*-YBCO), under conditions compatible with the silicon integration of complementary metal oxide semiconductor (CMOS) readout electronics; (ii) a low level of flicker noise (or 1/*f* noise) [21]. In addition, *a*-YBCO detector performances in the near infrared (NIR) are highly competitive. Butler et al. [20] reached a detectivity value $D^{*}\approx$ 10^8^ cm·Hz^1/2^·W^−1^ at ~3 kHz, but with a very slow response ($\tau_{th}\approx$ 35 ms) for suspended sandwich-like capacitor devices. The $\tau_{th}$ value was improved to 0.2–0.3 ms for a non-suspended structure but at the expense of a lower detectivity ($D^{*}\approx$ 10^7^ cm·Hz^1/2^·W^−1^ at ~3 kHz). More recently, state-of-the-art performances were evidenced by Cheneau et al. [22] for non-suspended small-size sandwich-like capacitor structures: a $D^{*}$ value larger than 10^7^ cm·Hz^1/2^·W^−1^ was extracted at 10 MHz with a thermal time constant as short as 16 ns. However, *a*-YBCO thin films exhibit a small direct current (DC) electrical conductivity ($\sigma_{DC}\approx 1.7$ mS/cm at room temperature [23]), leading to high access resistance of the sensing detector, which can be detrimental for further preamplifier impedance matching. Strongly non-linear current–voltage characteristic plots were also observed [23]. 

The calcium doping of oxygen-rich crystallized YBCO (with *x* > 0.7) has been reported in the framework of investigating the effect of the partial substitution of the Y^3+^ cation by Ca^2+^ (hereafter abbreviated Ca-YBCO), mainly on the superconducting properties (a moderate decrease in the critical temperature but a significant increase in the critical current density at 77 K [24,25,26,27]). It should be noted that the alkaline earth metal Ca can replace its companion Ba, which might offer a priori rather limited interest. However, Ca can also replace Y to produce Y_1-y_Ca_y_Ba_2_Cu_3_O_6+*x*_, which introduces a significant redistribution of the electric charges (sixfold local symmetry replaced by eightfold local symmetry [24]), from which a no less significant renewal of properties can be expected. In light of those considerations, we wished to consider the performances of radiation detectors based on Ca-YBCO compared to those of undoped YBCO-based devices that we develop routinely [21]. 

In Section 2 of this paper, we detail the film and device manufacturing steps and describe the experimental set-up for the device test at 850 nm wavelength. In Section 3, we review the surface microstructure, the physico-chemical properties, and the electrical transport properties of *a*-Ca-YBCO films. In Section 4, we focus our interest on the NIR pyroelectric response of radiation-sensing devices exploiting this material. In addition, the modeling of device responses is considered in view of understanding their unconventional features. An important point of reference will indeed be the original YBCO material and the devices fabricated from it. Finally, we conclude briefly in Section 5.

## 2. Experimental Details

### 2.1. Thin Film Deposition and Characterization

Both undoped and calcium-doped YBCO thin films were deposited by off-axis DC sputtering under a 33 Pa atmosphere of argon + oxygen with a 55% Ar/45% O_2_ flow ratio from cylindrical hollow targets (height 3 cm, diameter 5 cm, inserted in a copper housing). Deposition was performed at low temperature (<150 °C) with no further oxygenation step, thus typically leading to an amorphous semiconducting deoxygenated YBCO phase [28]. Two superconducting material targets, provided by CERACO GmbH (Ismaning, Germany), were used: one of the stoichiometric Y_1_Ba_2_Cu_3_O_6.9_ phase and the other one of the non-stoichiometric compound Y_0.9_Ca_0.1_Ba_2_Cu_3_O_6.9_ i.e., enriched in 10 at. % calcium. The deposition rate was 150 nm/h. Films of thicknesses ranging from 220 nm to 900 nm were deposited on 380 μm thick *p*-doped silicon substrates coated with a thermally grown 500 nm thick SiO*_x_* layer.

The topographical features of the film surface were investigated by atomic force microscopy (AFM) using a MultiMode 8-HR system in the PeakForce Tapping^®^ mode (Bruker, Palaiseau, France); first-order “flatten” image processing was applied to the acquired images. Surface morphology was also observed by scanning electron microscopy (SEM) using a Phenom XL desktop system (Thermo Fisher Scientific, Les Ulis, France) equipped with a secondary electron detector (SED). The SEM apparatus was equipped with an energy dispersive X-ray spectroscopy (EDS) detector for elemental composition identification.

### 2.2. Device Fabrication

**Figure 1:**
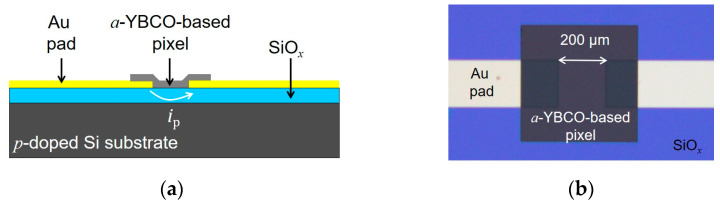
Typical planar pixel structure. (**a**) Cut view schematic; (**b**) Top view photography of a detector pixel fabricated on a SiO*_x_*//*p*-doped Si substrate. The distance between gold electrodes, as seen below the sensing film, is 200 µm.

Planar structures, as sketched in Figure 1a, were fabricated on *p*-doped silicon substrates. The technological process started with a bottom contact level, patterned by lift-off using a 10 nm thick titanium layer and a 180 nm thick gold layer deposited by e-beam evaporation on the substrate. An *a*-YBCO film or an *a*-Ca-YBCO film of thickness 500 nm was then deposited and patterned using standard optical lithography to define the active area (see Figure 1b).

### 2.3. NIR Optical Characterization

**Figure 2:**
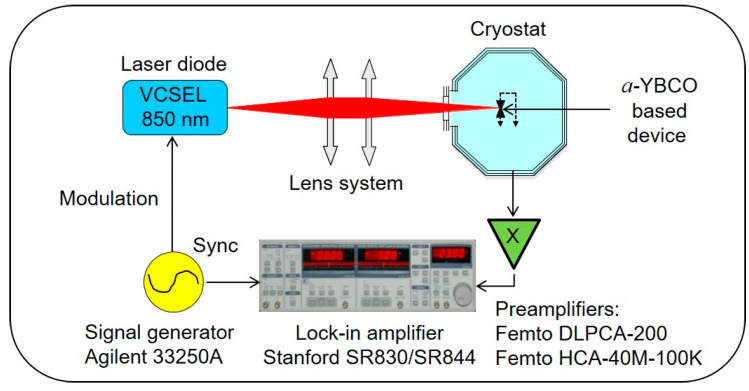
Experimental set-up for NIR tests. See text for details.

A schematic of the experimental set-up is shown in Figure 2. The device was fastened to the cold finger of a cryostat (Advanced Research Systems Inc., Macungie, PA, USA) equipped with a Suprasil^®^ optical window. The use of the cryostat metallic enclosure ensured efficient shielding of the device under test while keeping an operation temperature of ≈290 K. The unbiased device optical response was estimated by monitoring the delivered short-circuit current as a function of the modulation frequency f of an electronically modulated GaAs VCSEL laser diode emitting a few milliwatts at 850 nm wavelength (Honeywell HFE4080-322/XBA). The VCSEL output beam was collimated and focused onto the sample by a set of two convergent lenses, resulting in a 200 μm diameter spot (Gaussian beam waist). Due to the implementation of optical density filters and the consideration of the geometry of the sample, the device was effectively submitted to a power in the sub-milliwatt range. This effective incident power $P_{inc}$ was carefully deduced from a detailed geometrical description of the sample arrangement.

The device pyroelectric current $i_{p}$ was readout with a low input resistance trans-conductance preamplifier chosen according to the investigated bandwidth: (i) FEMTO^®^ DLPCA-200 (of adjustable gain, 500 kHz maximum bandwidth); (ii) HCA-40M-100K (0.1 V/μA gain, 40 MHz bandwidth). The voltage output of the preamplifier was synchronously detected at reference frequency f with a lock-in amplifier (Stanford Research Systems SR 830 up to 100 kHz, SR 844 above 25 kHz, allowing frequency overlap checking).

These lock-in amplifiers were also used to estimate the noise level thanks to their built-in “X noise” and “Y noise” channel functions. All measurements were performed by setting the lock-in low-pass filter time constant at τ=300 ms with slope at −6 dB/oct., providing an equivalent noise bandwidth ENBW =τ/4=0.83 Hz; for Gaussian noise, ENBW is the bandwidth of a perfect rectangular filter that passes the same noise intensity as the real filter. Due to the noise statistical nature that requires statistical treatment over a large number of noise samples, 2000 to 3000 measurement points were taken every 0.5 s to 1 s (for both X and Y channels). As every measurement took place while the previous measurement damping period was still active in the 75% (0.5 s rate) to 95% (1 s rate) range, there was a moving average procedure. The X and Y channels provided two uncorrelated estimations of the noise level measured in a 1 Hz bandwidth. As the measured noise current density $i_{nM}$ (expressed in A/Hz^0.5^) resulted from both the device noise current density $i_{nD}$ and the amplifying chain noise current density $i_{nA}$, the former was obtained by quadratic subtraction according to

(1) $$i_{nD}=\sqrt{i_{nM}^{2}\;-\;i_{nA}^{2}}.$$

$i_{nA}$ was obtained by disconnecting the device and leaving the preamplifier input open-circuited and shielded. 

From noise measurements, we extracted the noise equivalent power *NEP* (in a 1 Hz bandwidth) and specific detectivity $D^{*}$ values of the devices according to the following relationships:

(2) $$NEP=i_{nD}/R_{i},$$

(3) $$D^{*}=\sqrt{A}/NEP,$$

where $R_{i}$ is the device responsivity, expressed in A/W and defined as $R_{i}=i_{p}/P_{inc}$ and A is the effective detector area.

## 3. Pyroelectric Material Properties

**Figure 3:**
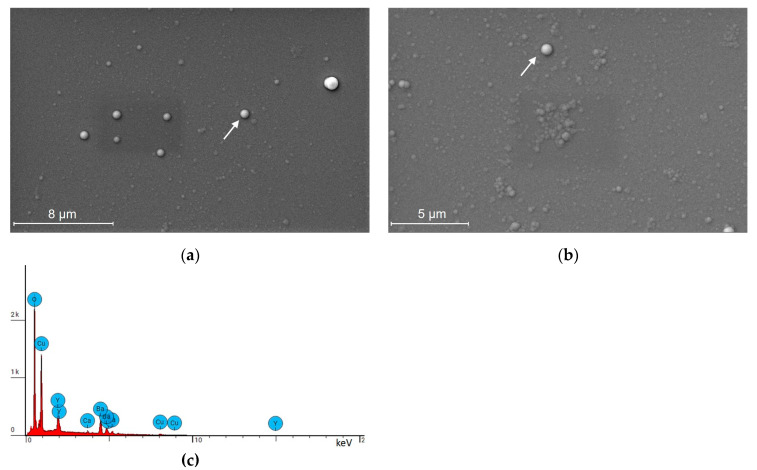
SEM study of a 900 nm thick *a*-Ca-YBCO film. (**a**) SEM image. (**b**) SEM image enlarged around the white dot indicated by the arrows. (**c**) EDS spectrum (mapping).

One example of the surface morphology of an *a*-Ca-YBCO film is shown in Figure 3a. The film consists of a uniform surface studded with clearly visible 0.7–1 µm diameter spherical dots (of surface density about 0.1/µm^2^). EDS analyses specifically carried out on these dots revealed that they were copper-rich and calcium-free precipitates. This could be explained by the presence of heterogeneous nucleation sites on the film surface, which could generate an electric field gradient favorable to growth [29]. By zooming in on the film surface, as displayed in Figure 3b, we observed two types of microstructure: clusters of grains isolated in a seemingly uniform matrix. EDS analyses specifically carried out on these clusters revealed that they were poor in calcium and rich in copper.

The EDS spectra obtained by mapping on various zones of Ca-doped YBCO films revealed the presence of Y, Ca, Ba, Cu, and O, confirming a correct element transfer from the target during deposition (see Figure 3c). The film cationic composition calculated with respect to the Y element is presented in Table 1. It is worth noting that the chemical compositions of the Ca-doped films were close to the nominal one (Y:Ca:Ba:Cu = 0.9:0.1:2:3) within the limits of accuracy of the technique. 

**Figure 4:**
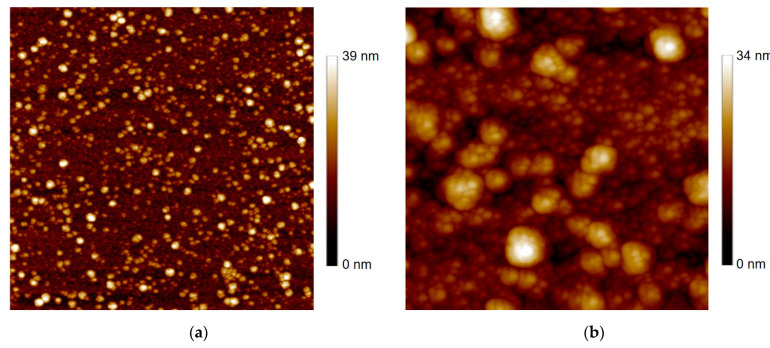
AFM images, with their *z*-axis scale, of a 900 nm thick *a*-Ca-YBCO film. (**a**) 10 × 10 µm^2^ area. (**b**) 2 × 2 µm^2^ area.

As illustrated in Figure 4a, the 10 × 10 µm^2^ area AFM image well revealed the granular structure of the *a*-Ca-YBCO film surface, with large grains or clusters isolated in a seemingly uniform amorphous matrix of smaller grains. This was already evidenced for *a*-YBCO films, exhibiting a matrix of nanostructured grains of 1 to 2 nm size [28]. The film in Figure 4a exhibited an rms roughness value $r_{AFM}=$ 5.5 nm rms. The 2 × 2 µm^2^ area image, displayed in Figure 4b, enlightens these characteristic features of the film surface morphology. The average grain size $d_{AFM}$ and the rms roughness $r_{AFM}$ values, as well as DC electrical properties (sheet resistance $R_{☐}$ and conductivity $\sigma_{DC}$) measured on patterned devices (see Section 4), are shown in Table 2. The $\sigma_{DC}$ value of Ca-doped YBCO films was 24 times lower than that of undoped YBCO films. As we dealt with a Ca-doped matrix with larger grains ($d_{AFM}\approx 50$ nm) and less uniform than in the undoped matrix consisting of nanograins ($d_{AFM}\approx 2$ nm), the electrical transport would ultimately not be improved in the Ca-doped films. This should be explored further in detail.

## 4. Pyroelectric Detector Performances: Results and Discussion

### 4.1. Device Optical Response

**Figure 5:**
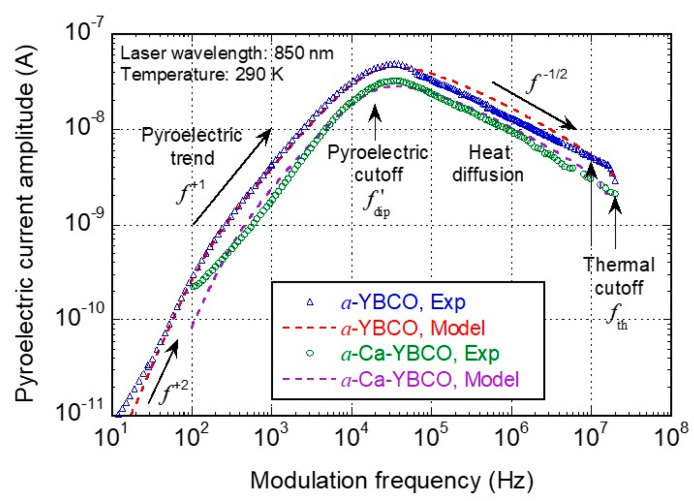
Experimental pyroelectric current (open symbols) and analytical model (red and purple dashed lines) as a function of the modulation frequency for undoped (blue triangles) and calcium-doped (green circles) YBCO-based planar devices. For the model, the parameter values were adjusted according to the maximum amplitude. The indicated frequencies ($f_{dip}^{\prime }$ and $f_{th}$) were extracted using the model (see text for details).

Figure 5 exhibits the output current responses for both undoped and calcium-doped *a*-YBCO-based devices. We observed similar trends for both devices:

- At very low frequencies (below ~100 Hz), a $f^{+2}$ behavior was visible only for the *a*-YBCO device that is related to a dipolar relaxation due to interfacial effects between the *a*-YBCO film and gold contact pads [23]. The *a*-Ca-YBCO device response was too noisy and could not be tested below 100 Hz;
- Between ~100 Hz and ~10 kHz, a low-frequency high-pass behavior was identified, which is typical of a pyroelectric response (capacitive effect occurring in this device structure at low frequency). The cutoff at ~20 kHz of this $f^{+1}$ behavior can be related to a second mechanism of dipolar relaxation in both the *a*-YBCO and *a*-Ca-YBCO films;
- Between 20 kHz and 80 kHz, a maximum current response was reached, with a lower amplitude for the *a*-Ca-YBCO device;
- Above ~100 kHz, the low-pass behavior, close to $f^{-1/2}$, was typical of heat diffusion through the substrate. Measurements were limited to 40 MHz due to the readout preamplifier cutoff.

These current output responses exhibited similar band-pass type trends as for traditional pyroelectric detectors [30] but with two unconventional slopes: the $f^{+2}$ slope at very low frequency and the $f^{-1/2}$ slope at higher frequencies. The cutoff frequency observed at 20 kHz cannot be obviously related to the thermal cutoff frequency, of which the values usually lie in the 0.1 to 10 Hz range for regular pyroelectric detectors [30]. These discrepancies led us to introduce substantial changes to the regular pyroelectric model, as discussed hereafter in Section 4.2. 

### 4.2. Analytical Model and Discussion

A pyroelectric detector is a capacitive device, the dielectric material of which is an yttrium–barium–copper oxide amorphous thin film in our case. The YBCO-based planar capacitor consists of the material comprised between the gold pads with the *p*-doped Si substrate acting as a floating counter electrode, as displayed in Figure 1. As already mentioned, the device, which has an inherent capacitive nature, does not need to be DC biased, and therefore the device has a reduced noise level (no resistive current). The results were analyzed using an analytical model [21], in the harmonic regime, for which various characteristic frequencies were considered. 

The pyroelectric mode is related to the modulation of the dielectric polarization P at frequency f. The absorption of incident radiation power $P_{inc}$ by the sensing pyroelectric material implies a material temperature variation ΔT. A thermal power balance in the sensing material allows the expression of ∆T, as follows:

(4) ΔT∆Pinc=ηGth(1+jf/fth) ,

where η is the power absorption coefficient, $G_{th}$ is the thermal conductance between the sensing area and the thermostat (at 290 K), and $f_{th}=1/2\pi \tau_{th}$ is the thermal cutoff frequency, with $\tau_{th}=C_{th}/G_{th}$ being the thermal time constant ($C_{th}$ is the thermal capacitance of the sensing volume).

Due to the temperature variation ∆T, a polarization variation ΔP occurs in the pyroelectric material, as follows:

(5) ΔPΔT=p jf/fdip1+ jf/fdip×jf/fdip′1+ jf/fdip′ ,

where p is the pyroelectric coefficient, and $f_{dip}$ and $f_{dip}^{\prime }$ are the two Debye’s dipolar relaxation frequencies [23]. The onset of polarization leads to the creation of surface electrical charges Q, which produce a pyroelectric current $I_{p}$ measured via the gold pads connected to the external electronic readout circuit: $I_{p}=j2\pi fQ=Aj2\pi f∆P$, where A is the effective sensing area. 

When combining the previous Equations (4) and (5), we obtain the following transfer function $H(f)=I_{p}/∆P_{inc}$ for the pyroelectric sensor as follows:

(6) $$H\left(f\right)=\frac{\eta Ap}{G_{th}}j2\pi f\times \frac{jf/f_{dip}}{1+jf/f_{dip}}\times \frac{jf/f_{dip}^{\prime }}{1+\;jf/f_{dip}^{\prime }}\times \frac{1}{1+jf/f_{th}}\times R_{el}\left(f\right)\;,$$

where $R_{el}\left(f\right)$ is the low-pass transfer function associated with the electronic readout circuitry.

Finally, to explain the unconventional high-frequency current response exhibited by our sensors, the frequency-dependent heat diffusion process through the substrate thickness must be taken into account. Following the model detailed in [21], the thermal contribution $1/G_{th}(1+jf/f_{th})$ to $H\left(f\right)$ in (6) should be replaced by

(7) $$1/G_{0}\left[1+{\left(f/f_{dif}\right)}^{1/2}+j{\left(f/f_{dif}\right)}^{1/2}+j(f/f_{th})\right]\;,$$

where $G_{0}$ is a constant and $f_{dif}$ is the diffusion cutoff frequency. 

The parameters used to fit the model response curves, as displayed in Figure 5, are given in Table 3. There is little difference between the two sensing materials, suggesting a minor role of calcium doping in this regard. However, this should be tempered by the fact that the detection volume of the *a*-YBCO film-based pixel is 74 times smaller than that of the *a*-Ca-YBCO film-based pixel. Thermal capacitance effects should be therefore considered for devices with the same geometry.

It is worth noting that the values of both dipolar relaxation cutoff frequencies are in line with the previous values obtained from undoped *a*-YBCO film dielectric measurements using the Debye model [23]. Whereas $f_{dip}$ observed at low frequency might be attributed to interfacial effects between the YBCO film and the gold contact pads, $f_{dip}^{\prime }$ observed at high frequency can be related to polarization inside the grains of the sensing film. 

Above $f_{dip}^{\prime }=20$ kHz, the thermal diffusion is localized inside the sensing film. As heat is first absorbed by the film before diffusing through the substrate, it is logical to find a diffusion cutoff frequency related to the film ($f_{dif}=100$ kHz) that is lower than the thermal cutoff frequency associated with the substrate ($f_{th}>10$ MHz). 

The thermal cutoff frequency might be higher than 10 MHz for the undoped YBCO device (resp. 20 MHz for the Ca-doped YBCO device), as our measurements, limited by the 40 MHz cutoff frequency of the preamplifier, were somewhat noisy in this frequency range.

### 4.3. Device Performances

**Figure 6:**
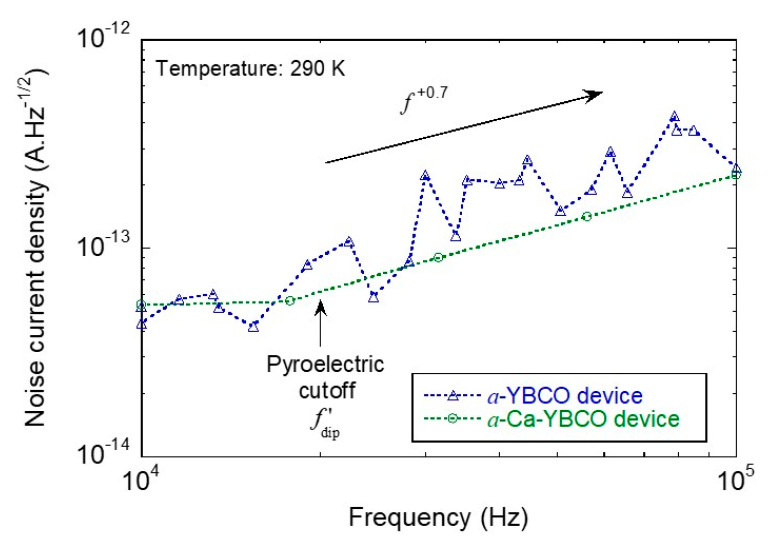
Device noise current density as a function of the measurement frequency for undoped (blue triangles) and calcium-doped (green circles) YBCO-based planar devices. Dashed lines are a guide for the eye.

Device noise current density values were recorded, as displayed in Figure 6. The first noticeable feature is the lower noise level for the Ca-doped YBCO device. Moreover, the $f^{+0.7}$ trend indicates the possibility of a hopping mechanism across a contact barrier as related to the non-linearity of current–voltage DC plots and the possible Schottky nature of the *a*-Ca-YBCO/metal contacts (as previously confirmed for *a*-YBCO/metal contacts [23]). 

In order to compare the device performances according to the undoped or Ca-doped nature of the sensing film material, we present *NEP*, specific detectivity $D^{*}$, and time constant $\tau_{th}$ results in Table 4, as deduced from the pyroelectric (at room temperature and 850 nm wavelength) and associated noise currents, and explained above in Section 2.3. It appears that the doped devices are less sensitive in terms of *NEP* but faster than their undoped counterparts, which leaves open the choice of the adequate device according to the envisaged application. It should be also noted that all of those performances (obtained from large size devices) fall short of reaching the performances we obtained from *a*-YBCO detectors with adequately tailored small-size trilayer structures [22]: e.g., such devices exhibit a $D^{*}$ value at 100 kHz, which is only about one order of magnitude lower than the theoretical limit value for uncooled detectors ($1.8\times 10^{10}$ cm·Hz^1/2^·W^−1^) [31].

It is also worth comparing the performance data of our detectors with those of LiTaO_3_ or PZT-based sensors commercially available or reported in the literature, as shown in Table 5. It should be noted that these detectivity values are given at their maximum, which is reached in the 3 Hz to 3 kHz modulation frequency region. It clearly appears that our YBCO-based devices, while exhibiting similar detectivity values at 10 kHz, are much faster by several orders of magnitude. 

## 5. Conclusions

To conclude, we have undertaken the study of amorphous calcium-doped YBCO material in view of its use as a sensitive film for the thermal detection of near infrared radiation. The films were deposited by sputtering on *p*-doped silicon substrates with a SiO*_x_* buffer layer. The films exhibited a granular surface, with copper-rich precipitates, and high DC electrical resistivity. *a*-Ca-YBCO/gold planar structure detector devices were fabricated and tested at 850 nm wavelength. Their response as a function of the modulation frequency of the incident beam was compared to that of detectors made from undoped *a*-YBCO. As for undoped *a*-YBCO, the response was that of a pyroelectric character, exhibiting a maximum at ~30 kHz. This response was correctly interpreted using an analytical model including dipolar relaxation, pyroelectric behavior, and heat diffusion in the substrate. *a*-Ca-YBCO devices are found to exhibit (i) a higher *NEP* value of ${3.6\times 10}^{-10}$ W·Hz^−1/2^ at 100 kHz; (ii) a higher specific detectivity with a $D^{*}$ value of ${7.5\times 10}^{7}$ cm·Hz^1/2^·W^−1^ at 100 kHz, and (iii) a faster response, with an 8 ns time constant instead of 16 ns for *a*-YBCO devices. *a*-Ca-YBCO is therefore revealed as an interesting candidate for the industrialization of fast infrared detector systems. 

## Figures and Tables

**Table 1:** Results of EDS analysis: cationic composition.

Type of *a*-YBCO Film (Thickness)	Atomic %				Cationic Composition	Nominal Composition
	Y	Ca	Ba	Cu		
Ca-doped (225 nm)	6.65	0.86	14.42	21.79	Y_0.89_Ca_0.11_Ba_1.92_Cu_2.90_	Y_0.9_Ca_0.1_Ba_2_Cu_3_
Ca-doped (900 nm)	6.73	0.77	15.7	23.16	Y_0.9_Ca_0.1_Ba_2.09_Cu_3.09_	Y_0.9_Ca_0.1_Ba_2_Cu_3_
Undoped (260 nm)	3.66	–	5.60	10.31	Y_1_Ba_1.84_Cu_2.82_	Y_1_Ba_2_Cu_3_

**Table 2:** Surface morphology features (as revealed in 2 × 2 µm^2^ area images) and room-temperature DC electrical properties of 900 nm thick films.

Type of *a*-YBCO Film	$r_{AFM}$ (nm rms)	$d_{AFM}$(nm)		$R_{☐}$ (MΩ/☐)	$\sigma_{DC}$ (mS/cm)
		Large Grains	Small Grains		
Ca-doped	4.3	270 ± 20	50 ± 10	158	0.07
Undoped	3	55 ± 5	2 ± 1	6.5	1.70

**Table 3:** Parameter values extracted from response model fitting.

Parameter	*a*-Ca-YBCO Device	*a*-YBCO Device
$f_{dip}$	300 Hz	130 Hz
$f_{dip}^{\prime }$	20 kHz	20 kHz
$f_{dif}$	100 kHz	100 kHz
$f_{th}$	20 MHz	10 MHz

**Table 4:** Performances of Ca-doped and undoped *a*-YBCO uncooled planar detectors at 850 nm wavelength.

*f* (Hz)	*NEP* (W·Hz^–1/2^)		$D^{*}$ (cm·Hz^1/2^·W^−1^)		$\tau_{th}$ (ns)	
	*a*-Ca-YBCO	*a*-YBCO	*a*-Ca-YBCO	*a*-YBCO	*a*-Ca-YBCO	*a*-YBCO
$10^{4}$	${1.7\times 10}^{-10}$	${7.6\times 10}^{-11}$	${2.6\times 10}^{8}$	${7.1\times 10}^{7}$	<8	<16
$10^{5}$	${6.0\times 10}^{-10}$	${3.6\times 10}^{-10}$	${7.5\times 10}^{7}$	${1.5\times 10}^{7}$		
$10^{6}$	$5.6\times 10^{-8\;\;\;}$	${2.5\times 10}^{-9\;\;\;}$	${8.0\times 10}^{5}$	${2.2\times 10}^{6}$		

**Table 5:** Performances of pyroelectric detectors published in the literature.

Reference	Material	f (Hz)	$D^{*}$ (cm·Hz^1/2^·W^−1^)	$\tau_{th}$ (ms)
[32]	LiTaO_3_	3	${2.5\times 10}^{8}$	200
[33]	LiTaO_3_	10	${3.0\times 10}^{8}$	150
[12]	LiTaO_3_	80	${1.7\times 10}^{8}$	5.8
[34]	PZT	80	${1.5\times 10}^{8}$	2
[20]	*a*-YBCO	$\sim {3\times 10}^{3}$	${1.0\times 10}^{7}$	0.2–0.3

## Data Availability

Data sharing is not applicable to this article.
